# A retrospective cohort study on the seizure risks and outcomes of children with acquired brain injury

**DOI:** 10.3389/fneur.2025.1629669

**Published:** 2025-09-10

**Authors:** Vivien W. Y. Li, Yuliang Wang, Winnie W. Y. Tso

**Affiliations:** ^1^Department of Paediatrics and Adolescent Medicine, Queen Mary Hospital and Duchess of Kent Children's Hospital, Hong Kong SAR, China; ^2^Department of Psychology, The University of Hong Kong, Hong Kong SAR, China; ^3^The Department of Paediatrics & Adolescent Medicine, The University of Hong Kong, Hong Kong SAR, China; ^4^The Hong Kong Children's Hospital, Hong Kong SAR, China

**Keywords:** pediatric acquired brain injuries, seizures, epilepsy, functional outcome, rehabilitation

## Abstract

**Background:**

The purpose of this study is to determine the prevalence, risk factors, and characteristics of seizures and epilepsy in children with acquired brain injury (ABI), and compare their outcomes with children with ABI but no seizures.

**Method:**

Basic demographic data, clinical features, brain injury severity, seizure and epilepsy characteristics, and functional and neurodevelopmental outcomes of children with ABI with follow-up of at least 2 years were reviewed. Logistic regression was performed to determine the risk factors for seizures.

**Results:**

The study included 82 children with ABI due to tumors, trauma, hypoxia, stroke, infection, and neuro-inflammatory disorders. There were 43 (52%) boys. The median age at diagnosis was 2.9 years and median follow-up interval was 5 years. A total of 27 (33%) children experienced seizures and 20 (24%) were diagnosed as having epilepsy. Risk factors for seizures included cortical brain injury (*p* = 0.013) and central nervous system (CNS) infection (*p* = 0.001). Among those with seizures, seven had acute seizures within 7 days of ABI. The median time of onset of epilepsy after ABI was 2 years, and five children had refractory epilepsy (RE) needing more than two anti-epileptics. The hazard ratio (HR) for epilepsy in those with cortical brain injuries and CNS infections were 4.582 (95% CI [1.83, 11.49], *p* = 0.001) and 4.796 (95% CI [1.568, 14.67], *p* = 0.006), respectively. HR for epilepsy onset in those who had post-stroke seizures were 4.467, 95% CI [1.575, 12.67], *p* =0.005). Most subjects demonstrated significant improvements in Karnofsky Performance Scale (KPS) scores following rehabilitation (*p* < 0.0001); however, a greater proportion of children with post-ABI seizures required special educational services (*p* = 0.025).

**Conclusion:**

Cortical brain injuries, CNS infection and post-stroke seizures significantly increase the risk of epilepsy in children with ABI. While functional improvements were observed after rehabilitation, children with post-ABI seizures more often required special educational support. The identification of risk factors for seizures, time to epilepsy onset, and the functional outcomes can guide subsequent management and counseling.

## Introduction

Acquired brain injury (ABI) occurs after birth and can result from trauma, tumor, infection, hypoxia, stroke, or immune-mediated disorders, but excludes sustained birth trauma, and congenital and degenerative causes (1). In children, ABI can disrupt brain development causing detrimental consequences for physical and mental health with long-lasting sequelae. Seizure is one of the most common sequelae that might occur in both the acute and subacute phases post-ABI. Studies have found that post-ABI seizures can manifest from 1 week to several decades following ABI, with a significant proportion of cases occurring within the first-year post-injury (2, 3). Despite recognizing the variability in timing, there remains a significant gap in understanding which specific ABI etiologies are associated with the highest risk of developing seizures. Majority of existing studies on seizure risks and neurodevelopmental outcomes in pediatric post-ABI looked at a single etiology (4–7), with only a few comparing the clinical outcomes between the different ABI etiologies (8, 9).

Existing literature reports a wide range of post-ABI epilepsy prevalence, spanning from 5 to 20% (10). This variability might be related to the different etiologies, types or severity of ABI, it might also be attributed to differing definitions of epilepsy across studies. According to the International League Against Epilepsy (ILAE) 2017 criteria, epilepsy is diagnosed after at least two unprovoked seizures more than 24 h apart, or a single unprovoked seizure with a high risk of recurrence. Hence, some studies define post-ABI epilepsy based solely on a single late post-traumatic seizure, reflecting the elevated likelihood of future seizures (11) but others would adopt the more conservative criterion of two unprovoked seizures (12, 13); patients were classified with brain tumor-related epilepsy (BTRE) if they had one or more unprovoked seizures at or after diagnosis of brain tumor (14); patients were only diagnosed with autoimmune encephalitis-associated epilepsy if seizures persist for more than 2 years after immunotherapy with no signs of ongoing encephalitis radiologically and biochemically (15), while others such as post-stroke epilepsy (16) would be classified at least two unprovoked seizures after acute phase of stroke. This inconsistency in operational definitions hampers accurate prevalence estimation and comparability across studies, highlighting a crucial gap in standardized diagnostic criteria for post-ABI epilepsy. Addressing this gap is essential as it can impact clinical management such as prescriptions of anti-epileptic medications for children at risk of post-ABI epilepsy.

Existing studies conducted on the adult population with traumatic brain injury demonstrated that post-traumatic epilepsy is a major driver of the neurological, emotional and occupational disability associated with traumatic brain injury (TBI) (17). Seizures cause secondary injury after acquired brain injuries, often exacerbating neurodegeneration, inflammation and blood–brain barrier dysfunction (18). Seizure also impairs neurological recovery after brain injury and had been found to independently associated with poor functional outcomes. For the young developing brain, the impact of seizure post brain injury might be even greater than the adult population as it significantly disrupts the developmental trajectory during the critical periods of brain development (19). Nevertheless, there are paucity of studies on the impact of post-ABI seizures on subsequent functional, neurodevelopmental, and cognitive outcomes in the pediatric population (13).

The primary aim of this study was to determine the prevalence of seizures and identify the associated risk factors in children with acquired brain injury (ABI), as well as to examine the characteristics of post-ABI seizures and epilepsy. Additionally, the study sought to assess the functional, cognitive, and neurodevelopmental outcomes in children following ABI, with a particular focus on those who developed seizures. We hypothesized that children with post-ABI seizures would experience worse outcomes across these domains. To achieve these objectives, the paper was organized to first provide a comprehensive overview on childhood ABI and its prevalence, we would present the seizure characteristics, alongside comparisons of functional and developmental outcomes between children with and without seizures. We would interpret the study findings in the context of existing literature and explore their implications for clinical management and rehabilitation strategies.

## Methods

A retrospective cohort study was performed by reviewing the medical records of children with ABI aged 0–18 years at the time of ABI diagnosis. Children throughout Hong Kong with ABI were referred to either the ABI programme under Duchess of Kent Children's Rehabilitation Hospital or the Hong Kong Children's Hospital. The ABI programme provides 4–8 weeks of multi-disciplinary intensive rehabilitation training. The programme is led by a pediatrician with a multi-disciplinary team of allied health professionals including physiotherapists, occupational therapists, speech therapists, clinical psychologists, dietitians, medical social workers, and nurses. The ABI programme aims to provide comprehensive and holistic care for children with ABI, starting at the initial phase, providing multidisciplinary rehabilitation, support for the families, provides caregivers' training, community and school re-integration as well as providing long-term follow-up.

Patients referred to the ABI programme between 1st January 2016 and 28th February 2022 were included in the study. Patients with birth asphyxia, with known genetic/metabolic disorders, or those lost to follow-up for more than 5 years were excluded.

Age at diagnosis, gender, etiology of ABI, Glasgow coma scale (GCS) at presentation, location, and severity of brain injury on first brain imaging, treatment modalities, use of prophylactic anti-epileptic drugs (AED) post-ABI, seizure characteristics, time to epilepsy onset from ABI diagnosis, first and latest scores on the Lansky play-performance scales (LPPS) or Karnofsky performance scales (KPS), the latest documented developmental quotient (DQ) or intelligent quotient (IQ), hearing impairment, vision impairment, motor deficits, psychiatric diagnosis, and special educational needs (SEN) were collected over a follow-up period of at least 2 years from diagnosis. For patients with seizures, use of prophylactic AEDs, timing of first seizure, seizure type, electroencephalography (EEG) findings, time to onset of epilepsy post-ABI, history of status epilepticus, epilepsy types, current AEDs, and epilepsy control were extracted.

Clinical severity of ABI at presentation was categorized into mild (GCS 14–15), moderate (GCS 9–13), and severe (GCS ≤ 8) (20, 21). The ABI locations were grouped into four categories: cortical, midline, infratentorial, and diffuse. Diffuse ABI included injuries in two or more regions of the brain. For cortical injury, the brain injury sites were further categorized according to cerebral lobe involvement: frontal, parietal, temporal, and occipital. Immediate complications of ABI were grouped into three categories according to the first CT brain findings: hydrocephalus, intracerebral bleeding, and cerebral oedema, with some having multiple complications.

Patients were defined as having acute seizures if seizures occurred within 1 week of intracranial surgery, traumatic brain injury, hypoxic encephalopathy, stroke, active central nervous system (CNS) infection, or autoimmune diseases (22). Seizure types were characterized according to the International League Against Epilepsy (ILAE) 2017 Classification (23). Patients were classified with brain tumor-related epilepsy (BTRE) if they had one or more unprovoked seizures at or after diagnosis of brain tumor (14); patients were classified with post-traumatic epilepsy if they had one or more unprovoked seizure 1 week after TBI (11), and only those who had seizures that persisted for more than 2 years after immunotherapy with no signs of ongoing encephalitis radiologically and biochemically would be classified as autoimmune encephalitis-associated epilepsy (15); otherwise patients were classified as having post-ABI epilepsy if they had two or more unprovoked seizures beyond 1 week after ABI diagnosis or if they were diagnosed with epilepsy syndrome. Patients with epilepsy characteristics were reported according to the ILAE 2017 position paper (24). Patients with seizures lasting for more than 30 min were defined as having status epilepticus, because irreversible neuronal injury was more likely to occur (25). Patients were considered to have refractory epilepsy (RE) if seizures were not controlled adequate trials of two tolerated and appropriately chosen antiepileptic drug (AED) regimens (26).

Functional performance status was assessed by LPPS in children aged 6–15 years and by KPS in children aged 16 years or older (27). The first LPPS/KPS post-ABI score was compared to the latest LPPS/KPS post-rehabilitation score for each individual, adjusted by follow-up time statistically. For children under 6 years, DQ was calculated using the Griffiths Mental Developmental Scales-Extended Revised (GMDS-ER) (28). For children aged 6–16 years, general IQ was estimated using the Hong Kong Wechsler Intelligence Scale for Children−4th Edition (WISC IV) (29). For children aged 16 years or older, IQ was estimated using the Wechsler Adult Intelligence Scale−3rd Edition Taiwan version (WAIS-III) (30). Development and cognitive level were defined by DQ/IQ score adjusted by follow-up time: ≥80 = normal; 71–79 = borderline low; ≤ 70 = global developmental delay (GDD) or intellectually disability (ID).

### Ethics

Ethics approval was obtained from the Institutional Review Board of the University of Hong Kong/Hospital Authority Hong Kong West Cluster and the Central Institutional Review Board of the Hospital Authority.

### Statistical analyses

Negative binominal logistic regression was performed to ascertain the effects of gender, ABI etiologies, brain injury sites, GCS scores at presentation, complications detected by first CT scan, and treatment types on the likelihood of having seizures, with follow-up time as a covariate. Quantile regression was used to compare the median age at diagnosis between seizure and non-seizure cases, and cox-regression was used to evaluate the hazard ratio (HR) of epilepsy onset. A generalized linear model was used to compare the outcomes of individuals who experienced seizures and those who never had seizures (fixed factor). The outcomes considered in the analysis included the initial and most recent LPPS/KPS scores, most recent DQ and IQ scores, requirement for special educational support, presence of motor deficits, hearing impairment, vision impairment, and diagnosis of psychiatric illness. Follow-up time was adjusted as a random factor in the model. Results were regarded as statistically significant for *p* < 0.05. Statistical analyses were performed using R 4.2.1 and figures were produced using GraphPad Prism 10.0 (GraphPad Software, Boston, Massachusetts, USA).

## Results

### Patient characteristics

**Figure 1:**
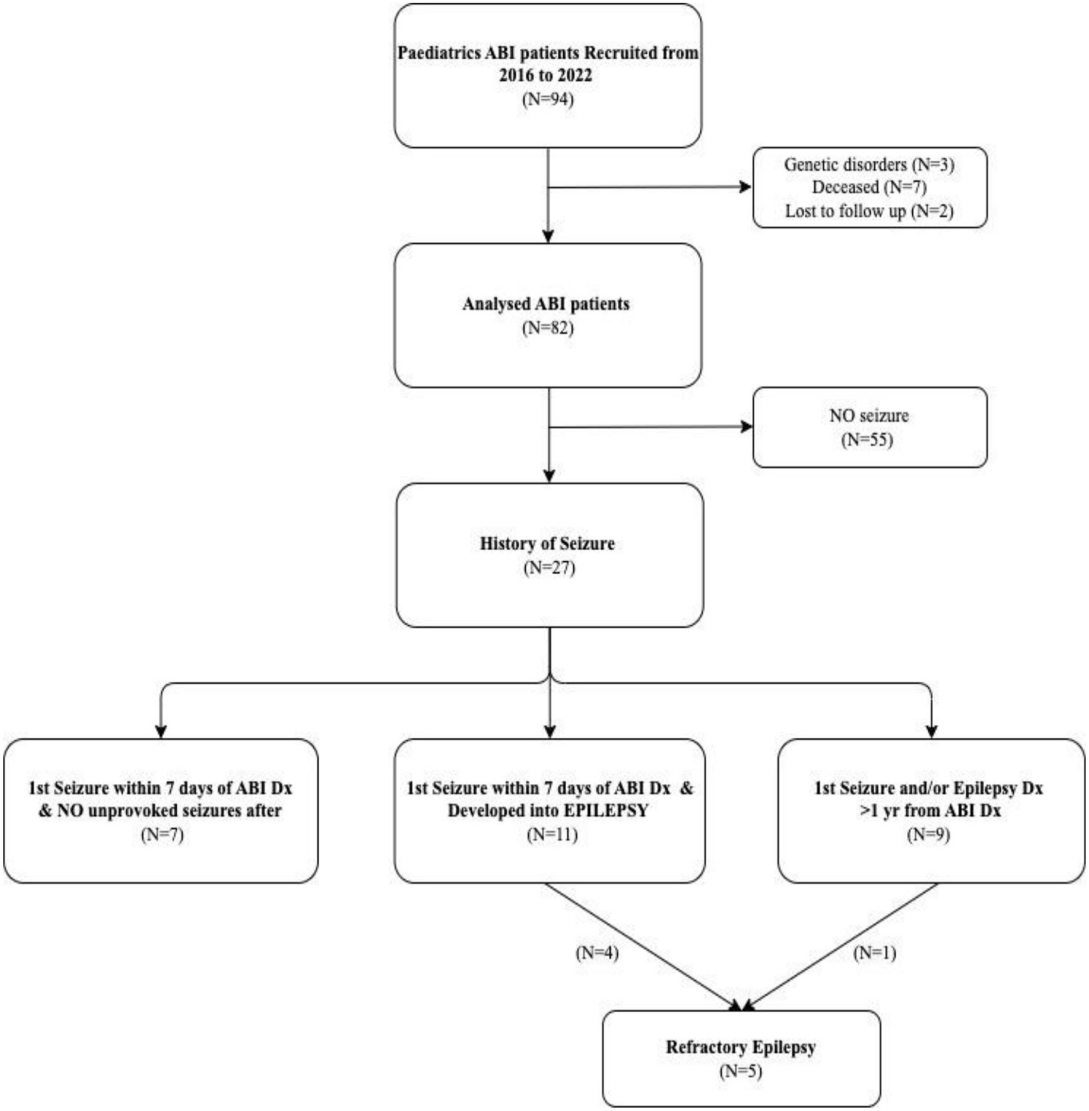
Flowchart of the study population. ABI, acquired brain injury; Dx, diagnosis; *N*; number.

A total of 94 children were referred to the ABI programme. After excluding children with genetic disorders (*n* = 3), those deceased during follow-up (*n* = 7), and those lost to follow-up (*n* = 2), a total of 82 post-ABI children were analyzed in our study (Figure 1 and Table 1). The median age at diagnosis was 2.9 years and median follow-up was 5 years. There were 43 (52%) boys and 39 (48%) girls. The causes for ABI were brain tumors (53 patients, 65%), followed by hypoxic brain injuries (14, 17%), Stroke (6, 7%), CNS infection (4, 5%), traumatic brain injuries (TBI; 4, 5%), and immune-mediated brain injury (1, 1%). Site of ABI lesions were mostly in infratentorial (25 patients, 30%) and cortical regions (24, 29%), followed by diffuse ABI (21, 26%) and midline lesions (12, 15%). At ABI diagnosis, 62 (76%) patients had GCS scores of 14–15, 6 (7%) had GCS scores of 9–13, and 14 (17%) had GCS scores of 3–8. At ABI diagnosis, 57 (70%) patients had one or more radiological complications including cerebral oedema, intracerebral bleeding, and/or hydrocephalus. Treatments for ABI included prophylactic AED (25 patients, 30%), intracranial surgeries (57, 70%), cardio-pulmonary resuscitation (CPR; 7, 8%), and extracorporeal membrane oxygenation (ECMO; 8, 10%). Treatments for those with brain tumors included chemotherapy or targeted therapy (41 patients, 50%), focal brain irradiation (18, 22%), cranio-spinal irradiation (15, 18%), and stem cell transplant (7, 9%).

**Table 1:** Demographics and intervention given to children with ABI.

**Variable**	**Number of patients (%)**				***p*** **-Value (Adj FU)**	
	**Total** ***N*** = **82**	**Seizures** ***N*** = **27 (33%)**		**No seizures** ***N*** = **55 (67%)**		
**Age at diagnosis**						
• Median age (years)	2.9 (0–18.7)	1.8 (0–13.9)		4.0 (0–18.7)	0.062	
• Mean age (years)	4.5 (0–18.7)	3.2 (0–13.9)		5.0 (0–18.7)	0.071	
**Follow-up time**						
• Median duration (years)	5.0 (2-14)	6.0 (2-14)		4.0 (2-10)	0.013^Λ^	
• Mean duration (years)	5.2 (2-14)	6.0 (2-14)		4.6 (2-10)	0.016^Λ^	
**Gender**						
• Female	39 (48%)	12 (44%)		27 (49%)	0.375	
• Male	43 (52%)	15 (56%)		28 (51%)	0.375	
**Causes of ABI**						
• Brain tumors	53 (65%)	14 (52%)		39 (71%)	0.099	
° ATRT	2	0		2	/	
° Ependymoma	6	0		6	/	
° Germ cell tumor	10	0		10	/	
° Glioblastoma multiforme	3	2		1	/	
° Low-grade glioma	11	2		9	/	
° High-grade glioma	2	1		1	/	
° Medulloblastoma	13	5		8	/	
° Others^#^	6	4		2	/	
• Hypoxic brain injury	14 (17%)	4 (15%)		10 (18%)	0.747	
• Traumatic brain injury	4 (5%)	2 (7%)		2 (4%)	0.396	
• Stroke^&^	6 (7%)	3 (11%)		3 (5%)	0.083	
° Arterial stroke	5	3		2	/	
° Venous stroke	1	0		1	/	
• CNS infection	4 (5%)	**4 (15%)** ^**^		0	0.001^**^	
• Immune-mediated brain injury	1 (1%)	0		1 (2%)	N/A	
**GCS at presentation**						
• Mild (GCS: 14–15)	62 (76%)	21 (78%)		41 (75%)	0.703	
• Moderate (GCS: 9–13)	6 (7%)	1 (4%)		5 (9%)	0.233	
• Severe (GCS: ≤ 8)	14 (17%)	5 (18%)		9 (16%)	0.754	
**Sites of brain injury**						
• Cortical	24 (29%)	**13 (48%)** ^*^		11 (20%)	0.013^*^	
° Frontal	5	3		2	/	
° Parietal	4	1		3	/	
° Temporal	5	3		2	/	
° Occipital	1	1		0	/	
°≥2 cortical regions involved	9	5		4	/	
• Midline	12 (15%)	3 (11%)		9 (16.5%)	0.389	
• Infratentorial	25 (30%)	5 (19%)		**20 (36.5%)** ^**^	0.007^**^	
• Diffuse	21 (26%)	6 (22%)		15 (27%)	0.505	
**Complications on first CT brain**						
• ≥1 complication(s)	57 (70%)	17 (63%)		40 (73%)	0.307	
° Cerebral oedema	6 (7%)	2 (7%)		4 (7%)	0.984	
° Intracerebral bleeding	12 (15%)	5 (19%)		7 (13%)	0.531	
° Hydrocephalus	25 (30%)	6 (22%)		19 (35%)	0.210	
°≥2 complications	14 (17%)	4 (15%)		10 (18%)	0.747	
• No complications on CT brain	24 (29%)	10 (37%)		14 (25%)	0.307	
• No brain imaging done	1 (1%)	0		1 (2%)	/	
**Treatments** ^##^						
• Prophylactic AED	25 (30%)	AS	AS + E	17 (31%)	0.406	0.918
		4 (15%)	8 (30%)			
• Intracranial surgery	57 (70%)	18 (67%)		39 (71%)	0.715	
• CPR	7 (8%)	2 (7%)		5 (9%)	0.634	
• ECMO	8 (10%)	3 (11%)		5 (9%)	0.732	
• Chemotherapy/Target therapy	41 (50%)	10 (37%)		31 (56%)	0.657	
• Radiotherapy	33 (40%)	6 (22%)		**27 (49%)** ^**^	0.009^**^	
° Focal brain irradiation	18 (22%)	0		**18 (33%)** ^***^	<0.001^***^	
° Cranio-spinal irradiation	15 (18%)	6 (22%)		9 (16%)	0.840	
• SCT	7 (9%)	1 (4%)		6 (11%)	0.655	

^#^The “Others” subtype including primary brain tumor: craniopharyngioma, ganglioglioma, low grade glioneuronal tumor, high grade neuroepithelial tumor, and primitive neuro-ectodermal tumors; and secondary brain tumor: brain metastasis from neuroblastoma.

^##^Patients with ABI can receive more than one treatment modality. AS included four patients who had acute seizures despite the use of prophylactic AED; AS +E included eight patients who had prophylactic AED but had acute seizures only, acute seizures and epilepsy later, and those who had epilepsy more than 1 year after ABI.

^&^Three patients had arterial ischemic stroke experienced seizures, one patient had arterial ischemic stroke without seizures seizures; one patient had arterial haemorrhagic stroke without seizures; one patient had venous ischemic stroke without seizure.

Statistically significant (^*^p < 0.05; ^**^p < 0.01; ^***^ *p* < 0.001). Bold italic indicates statistical significance (*p* < 0.05).

^Λ^*p*-value for follow-up duration and, therefore not adjusted by follow-up time itself.

ABI, acquired brain injury; AED, anti-epileptic drugs; Adj FU, adjusted for follow-up time statistically; AS, acute symptomatic seizures within 7 days of ABI; ATRT, atypical teratoid rhabdoid tumors; CNS, central nervous system; CPR, cardio-pulmonary resuscitation; E, epilepsy; ECMO, extracorporeal membrane oxygenation; GCS, Glasgow coma scale; *N*, number; SCT, stem cell transplant.

### Seizures and epilepsy in children with ABI

Among the children with ABI, 27 (33%) experienced at least one seizure after ABI. The median age at seizure onset was 1.8 years. The main causes of ABI in those with post-ABI seizures were brain tumor (14 patients, 52%), CNS infection (4, 15%), hypoxic brain injury (4, 15%), stroke (3, 11%), and TBI (2, 7%). In the study cohort, seizures occurred in all four patients with CNS infections (three had neonatal meningitis including one with Group B streptococcal (GBS) meningitis complicated by right cerebral hemorrhage, one with *Escherichia coli* meningitis complicated by right-sided arterial ischemic stroke, one with GBS meningitis; and one had epidural empyema), followed by 50% of patients with TBI, 50% with stroke (three had non-neonatal arterial ischemic stroke), 28% with hypoxic brain insult, and 26% with brain tumors. Approximately half of patients (13, 48%) had cortical brain injuries, followed by 6 (22%) with diffuse ABI, 5 (19%) with infratentorial lesions, and 3 (11%) with midline lesions. At ABI diagnosis, 21 (78%) patients had a GCS score of 14–15 and 17 (63%) patients had one or more radiological complications (Table 1).

Among the 18 patients with acute seizures post-ABI, 12 (66%) had seizures at presentation due to underlying ABI, 1 (6%) had TBI and severe hypernatraemia, and 5 (28%) had seizures within 1 week post-intracranial surgery. Regarding the site of ABI, 8 (44%) patients had cortical lesions, 3 (17%) had midline lesions, 2 (11%) had infratentorial lesions, and 5 (28%) had diffuse ABI. Thirteen (72%) patients had focal seizures, 4 (22%) had generalized tonic-clonic seizures (GTCS), and one had unknown seizure type captured on the EEG. Other focal seizure details are shown in Table 2. Seven patients had acute seizures without recurrence and 11 subsequently developed epilepsy.

Table 2Post-ABI acute seizures and epilepsy characteristics.

**Table:** 

**Variable**	**Post-ABI seizures *n* = 27 (%)**		
	**AS and no recurrence (*n* = 7)**	**AS and Epilepsy (*n* = 11)**	**Epilepsy >1 year post-ABI (*n* = 9)**
History of status epilepticus (SE)	1	3^Λ^	1
**Underlying ABI etiologies**			
• Brain tumor	3	6	5
• CNS infection	1	3	0
• Stroke	0	2	1
• Hypoxic brain injury	1	0	3
• Traumatic brain injury	2	0	0
**Sites of brain injury**			
• Cortical	2	6	5
• Midline	2	1	0
• Infratentorial	1	1	3
• Diffuse	2	3	1

**Table:** 

**Precipitating factors of acute seizures**	**AS (*n* = 18) (67%)^ΛΛ^**	**Epilepsy (*n* = 20) (74%)^ΛΛΛ^**
• Within 7 days post-intracranial surgery	5(28%)	/
° Brain tumor	3	
° CNS infection	1	
° Stroke	1	
• Electrolyte disturbance^#^	1 (6%)	
• Acute symptomatic seizures due to ABI	6 (33%)	
° CNS infection	3	
° Stroke	1	
° Hypoxic brain injury	1	
° Traumatic brain injury	1	
• Presented with seizures at BT diagnosis	6 (33%)	
**Seizure types**		
• Focal	13 (72%)	16 (80%)
° Focal (not specified awareness)	4 (22%)	0
° Focal aware	5 (28%)	7 (35%)
° Focal impaired awareness	4 (22%)	9 (45%)
• Generalized	4 (22%)	4 (20%)
° Tonic-clonic	4 (22%)	4 (20%)
• Unknown	1 (6%)	0
**Focal seizure onset** ^##^		
• Motor	11 (61%)	12 (60%)
° Clonic	10 (56%)	6 (30%)
° Tonic	1 (5%)	2 (10%)
° Automatism	0	1 (5%)
° Mixed	0	3 (15%)
• Non-motor	2 (11%)	3 (15%)
° Emotional	2 (11%)	2 (10%)
° Cognitive	0	1 (5%)
• Motor and non-motor mixed	0	1 (5%)
**Epilepsy onset time from ABI**		
• Median onset time (years)	/	2.0 (0–7.3)
**Epilepsy interictal EEG pattern**		
• Epileptiform discharge	/	15 (75%)
° Correlating to ABI regions		12 (60%)
° Over alternative brain regions		3 (15%)
• No epileptiform discharge		4 (20%)
• No EEG performed		1 (5%)
**Epilepsy control**		
• Seizure free >12 months	/	10 (50%)
• Seizure free >1 to < 12 months		5 (25%)
• Monthly		2 (10%)
• Daily		3 (15%)
**Refractory epilepsy**	/	5 (25%)
• Medical treatment with ≥2 AEDs		4 (20%)
• Epilepsy surgery		1 (5%)

^#^Electrolyte disturbance for index patient was due to hypernatraemia (Highest serum sodium level was 194 mmol/L).

^##^Seizure onset (for those with focal seizures/epilepsy only)—all generalized onset seizure in our study were generalized tonic-clonic seizures.

^Λ^Two patients had SE at both diagnoses of acute seizures and epilepsy; one had acute seizures; but only had SE on the diagnosis of post-ABI epilepsy subsequently.

^ΛΛ^Acute seizures (*n* = 18) included acute seizure types in patients with acute seizures only and acute seizures that developed into epilepsy.

^ΛΛΛ^Epilepsy (*n* = 20) included epilepsy types in patients with acute seizures that developed into epilepsy, and those who developed epilepsy >1 year after ABI.

ABI, acquired brain injuries; AED, anti-epileptic drugs; AS, acute seizures within 7 days of ABI; BT, brain tumors; CNS, central nervous system; EEG, electroencephalogram; *N*; number: SE, status epilepticus.

Out of the 82 patients, 20 (24%) were diagnosed with epilepsy post-ABI, whereas nine experienced their first seizure or developed epilepsy more than 1 year after ABI. The median epilepsy onset time after ABI was 2 years (range = 0–7.3 years). Among the 11 patients with BTRE, six presented with seizures at the time of diagnosis, two experienced seizures due to brain tumor recurrence at 1.7 years and new leptomeningeal spread at 7.3 years, respectively, and three had unprovoked seizures more than 1 year after the initial diagnosis. Among the three patients with CNS infection who developed epilepsy, two of them had acute status epilepticus and then remained seizure free for 2.5 years, but status epilepticus subsequently recurred, whereas the other patient developed electrical status epilepticus during slow-wave sleep (ESES) 3 years after the initial CNS infection. Among the three patients with non-neonatal arterial ischemic stroke who developed epilepsy, two had acute seizures and then remained seizure free, but developed epilepsy after 1.1 and 3.9 years, respectively, whereas the other patient developed epilepsy after 1.2 years. Three patients with hypoxic brain injury developed epilepsy after 2–3 years. Regarding the ABI site in those with epilepsy, 11 (55%) had cortical lesions, 1 (5%) had midline lesions, 4 (20%) had infratentorial lesions, and 4 (20%) had diffuse ABI. The majority of post-ABI epilepsy patients (16, 80%) had focal epilepsy, whereas 4 (20%) had generalized epilepsy (see Table 2 for more details). Fifteen (75%) epilepsy patients had epileptiform discharges on their EEG, of which 12 (60%) had epileptiform discharges correlating to ABI-involved sites and 3 (15%) had epileptiform discharges over alternative brain regions. At the latest follow-up, 10 out of these 20 patients (50%) were seizure free for more than 1 year, 5 (25%) were seizure free for more than 1 month but < 1 year, 2 (10%) had monthly seizures, and 3 (15%) had daily seizures. In the five patients (25%) diagnosed with refractory epilepsy (RE), four were female and one was male with age at ABI diagnosis ranging from neonatal to 7.3 years. Four of these patients had acute seizures prior to RE and one developed RE 2.3 years after ABI. One patient had ESES, whereas the other four patients had no history of status epilepticus. Regarding the brain injury site, one was cortical, two were diffuse, one infratentorial, and one was midline. The ABI etiologies also differed between RE patients, including two with brain tumors, two with stroke, and one with CNS infection. Four patients were taking more than two AEDs, and one patient underwent epilepsy surgery and was on one AED with good seizure control (Supplementary Table S1). All five patients shared similar parenchymal loss or encephalomalacia in frontal and/or temporal regions, as evident on the latest MRI brain imaging (Supplementary Table S2).

### Risk factors predictive of post-ABI seizures

**Figure 2:**
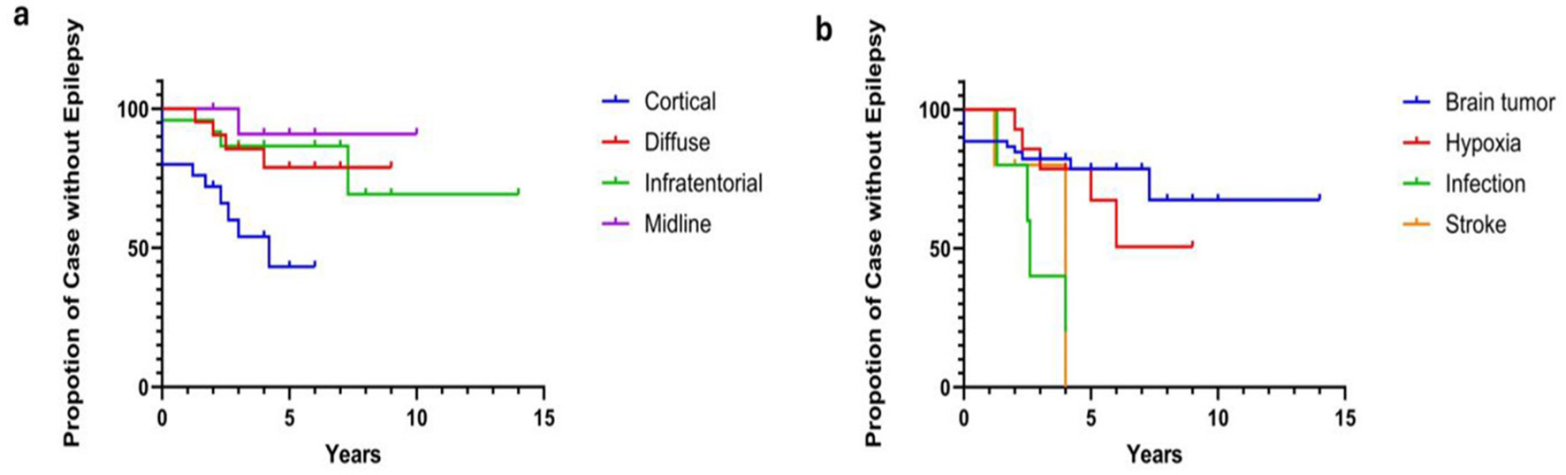
**(a, b)** Cox regression of brain injury sites and ABI etiologies on epilepsy outcome. Cox regression analysis demonstrated a significant difference in the trends observed in the Kaplan-Meier curves for distinct brain injury regions predicting epilepsy onset, χ 3 = 12.88, *p* = 0.0049 **(a)**, as well as for various types of brain injury predicting epilepsy onset, χ 3 = 10.22, *p* = 0.0168 **(b)**. Specifically, the Hazard Ratio (HR) of epilepsy onset for brain injuries in the cortical area is 4.582 [95% CI (1.83, 11.49), *p* = 0.001]. For CNS infections, the HR is 4.796 [95% CI (1.568, 14.67), *p* = 0.006]. For stroke patients, the HR for epilepsy is 4.467 [95% CI (1.575, 12.67), *p* = 0.005].

Logistic regression analysis showed the risk factors for post-ABI seizures were cortical brain injuries (*p* = 0.013) and CNS infection (*p* < 0.001), after adjusting for the length of follow-up. The relative risk (RR) of seizure was 1.45 [95% CI (1.17, 1.71), *p* < 0.001] for cortical ABI; and 1.94 [95% CI (1.87, 2.01), *p* = 0.001] for CNS infections, suggesting those with cortical ABI are of 50% higher risks in developing seizures compared to those with non-cortical injuries, and up to almost two-fold increased risk of seizures for those with CNS infection compared to those who had other ABI etiologies. Cox regression analysis demonstrated a significant difference in the trends observed in the Kaplan–Meier curves for distinct brain injury regions predicting epilepsy onset, χ3 = 12.88 (*p* = 0.0049; Figure 2a), and for various types of brain injury predicting epilepsy onset, χ3 = 10.22, (*p* = 0.0168; Figure 2b). Specifically, for brain injuries, the HR of epilepsy onset in the cortical area was 4.582 [95% CI (1.83, 11.49), *p* = 0.001], whereas for CNS infections, the HR was 4.796 [95% CI (1.568, 14.67), *p* = 0.006]. Thus, this means that individuals with cortical brain injuries as well as those with CNS infections, were four times more likely to develop epilepsy than those without such injuries. The comparison of seizure risk between stroke patients and other ABI patients did not reach statistical significance (*p* = 0.083). All three stroke patients who experienced seizures subsequently developed epilepsy. Among cases who experienced post-ABI seizure, stroke patients are more likely to develop epilepsy, HR = 4.467 [95% CI (1.575, 12.67), *p* = 0.005].

Twenty out of 25 children with infratentorial brain tumors never had seizures, hence, these patients (*p* = 0.007) were less likely to develop seizures. Among the 53 patients with brain tumors, 33 had radiotherapy [15 had cranio-spinal irradiation (CSI) and 18 had focal brain irradiation]. In those receiving radiotherapy, six patients with CSI had seizures, while none of 18 patients with focal brain irradiation developed seizures (*p* < 0.001).

In our cohort, prophylactic AEDs [including levetiracetam (10–25 mg/kg/day), phenytoin (5–20 mg/kg/day), valproate (10–30 mg/kg/day), or phenobarbitone (5 mg/kg/day)] were given at different dosages and durations to 25 patients (30%) at ABI diagnosis (Supplementary Table S3). Among the 55 patients who never had seizures, 17 patients (31%) had prophylactic AED; and among the 27 patient who had post-ABI seizures, 8 (30%) had also had prophylactic AED. Out of the eight patients who had post-ABI seizures, two had acute seizures within 7 days of ABI and no recurrence thereafter, two had acute seizures and developed epilepsy 1–3 years later, and four on prophylactic AED for 1 week to 11 months developed epilepsy 2–4 years later. Use of prophylactic AED was not statistically significant in reducing the risk of acute seizures (*p* = 0.406) or post-ABI seizures (*p* = 0.918).

### Outcomes of post-ABI children

**Figure 3:**
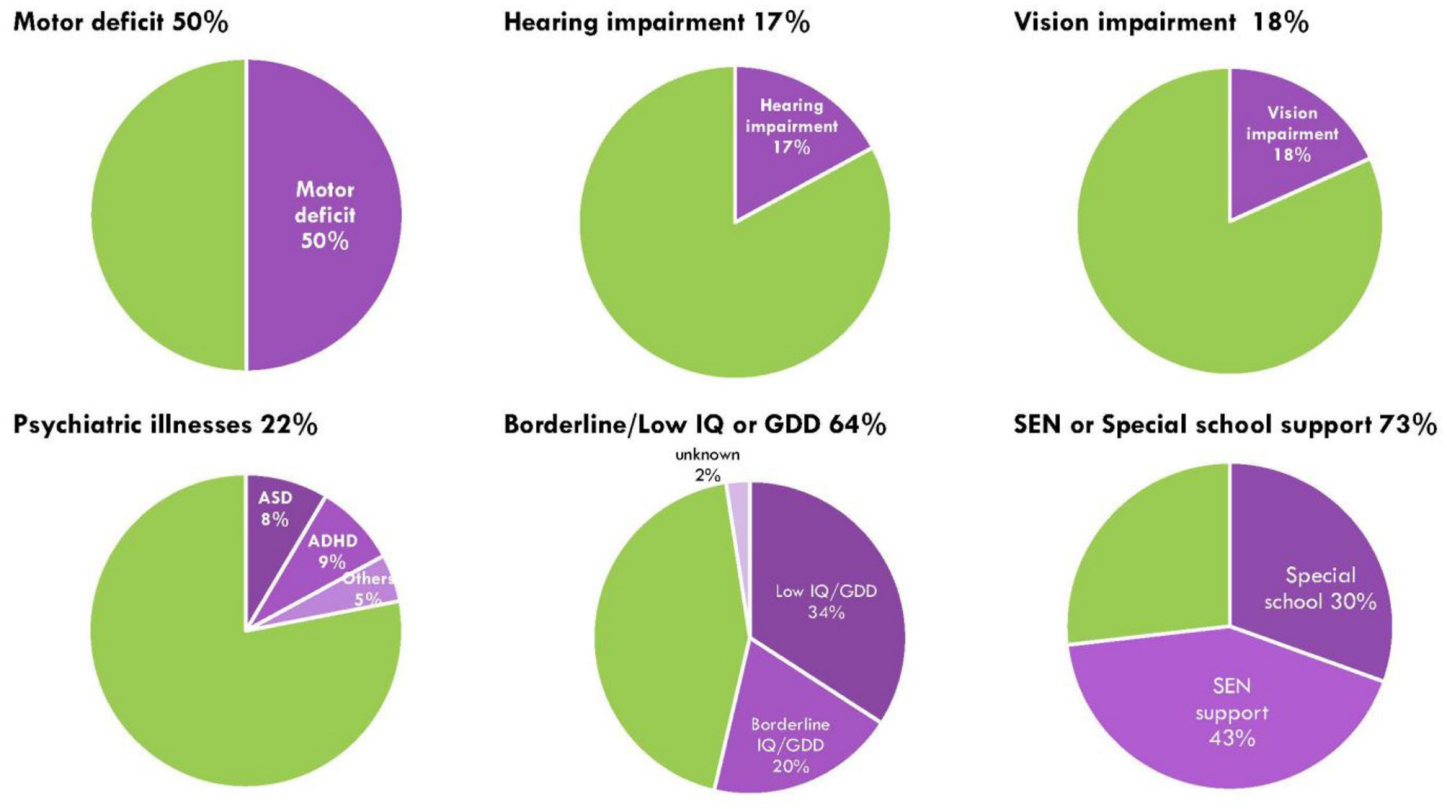
Sensorimotor, neurodevelopmental and psychiatric impairment in post-ABI children. Green represents proportion of children no deficits or impairment; purple shades represent proportion of children with either motor impairment, hearing impairment, vision impairment, psychiatric illnesses, cognitive or developmental delay, and those with special education needs or needing special school support.

All children completed 4–8 weeks of intensive rehabilitation training under the ABI program. The median first and latest LPPS/KPS scores were 20 (range = 10–90) and 90 (range = 30–100), respectively, indicating significant improvements in their functional outcomes following the rehabilitation period (*p* < 0.01). The median difference between the first and latest LPPS/KPS score was 70 (range = −10 to 90) for all patients, while the difference scores for the seizure group and non-seizure group were 60 (range = −10 to 80) and 70 (range = 0–90), respectively. Patients in the seizure group had a worse KPS score at follow-up compared to non-seizure group, however the score differences between the two groups was not significant (*p* = 0.228). Overall, less than half of patients (36, 44%) had normal DQ/IQ, 16 (20%) had borderline DQ/IQ, and 28 (34%) had GDD or ID. There were no significant differences in the developmental and cognitive outcomes between seizure and non-seizure groups, although 10 patients (37%) in the seizure group had normal DQ/IQ compared to 26 (47%) in the non-seizure group (*p* = 0.639), 5 (18%) in the seizure group had borderline DQ/IQ compared to 11 (20%) in the non-seizure group (*p* = 0.839), and 11 (41%) in the seizure group had GDD or ID compared to 17 (31%) in the non-seizure group (*p* = 0.507). Among the 27 children with post-ABI seizures, 23 (85%) required SEN support compared to 37 out of 55 (67%) who never had seizures (*p* = 0.025). At their latest follow-up, 41 (50%) had motor deficits, 14 (17%) had hearing impairments, 15 (18%) had vision impairments, and 18 (22%) had psychiatric illnesses, of which 7 (8.5%) had autism spectrum disorder, 7 (8.5%) had attention deficit hyperactivity disorder, and 4 (5%) had post-traumatic stress disorder, adjustment disorder, organic brain syndrome, and selective mutism, respectively (Figure 3). There were no differences in the proportion of children with motor deficits, hearing or vision impairments, or psychiatric illnesses between seizure and non-seizure groups (Table 3). For the comparison of subscale IQ and DQ scores, please see Supplementary Table S4.

**Table 3:** Outcomes in children with ABI.

**Variable**	**Number of patients (%)**			***p*-Value (adj FU)**
	**Total** ***n*** = **82**	**Seizures** ***N*** = **27 (33%)**	**No seizures** ***N*** = **55 (67%)**	
**Functional performance outcome**				
• Median First LPPS/KPS score	20 (10-90)	20 (10-90)	20 (10-80)	1.00
• Median Latest LPPS/KPS score	90 (30 −100)	80 (0 −100)	90 (30 −100)	0.137
• Median LPPS/KPS score difference	70 (−10–90)	60 (−10–80)	70 (0–90)	0.228
**Developmental and cognitive outcome**				
• Normal (DQ/IQ ≥ 80)	36 (44%)	10 (37%)	26 (47%)	0.639
• Abnormal (DQ/IQ ≤ 79)	44 (54%)	16 (59%)	28 (51%)	0.639
° Borderline (DQ/IQ: 71–79)	16 (20%)	5 (18%)	11 (20%)	0.839
° GDD/ID (DQ/IQ ≤ 70)	28 (34%)	11(41%)	17 (31%)	0.507
• No formal Assessment	2 (2%)	1 (4%)	1 (2%)	
**Schooling**				
• Mainstream school	22 (27%)	4 (15%)	**18 (33%)** ^*^	0.025^*^
• Special education support	60 (73%)	**23 (85%)** ^*^	37 (67%)	0.025^*^
° SEN in mainstream school	35(43%)	10 (37%)	25 (45%)	0.655
° Special school^#^	25(30%)	13 (48%)	12 (22%)	0.099
Motor deficit	41 (50%)	14 (52%)	27 (49%)	0.786
Hearing impairment	14 (17%)	4 (15%)	10 (18%)	0.921
Vision impairment	15 (18%)	8 (30%)	7 (13%)	0.063
Psychiatric illness	18 (22%)	4 (15%)	14 (25%)	0.542
• ASD	7 (8.5%)	3 (11%)	4 (7%)	/
• ADHD	7 (8.5%)	1 (4%)	6 (11%)	/
• Other^##^	4 (5%)	0	4 (7%)	/

^*^Statistically significant *p* < 0.05 comparing seizure and no seizure group, adjusted for follow-up time. Bold italic indicates statistical significance (*p* < 0.05).

^#^Special schools for intellectually disabled, physically impaired, or visually impaired.

^##^Other Psychiatric illnesses included post-traumatic stress disorder (PTSD), adjustment disorder, organic brain syndrome, and selective mutism.

ABI, acquired brain injury; ADHD, attention deficit hyperactivity disorder; Adj FU, adjusted for follow-up time statistically; ASD, autistic spectrum disorder; DQ, development quotient; GDD, global developmental delay; ID, intellectually disability; IQ, intelligence quotient; KPS, Karnofsky Performance Score; LPPS, Lansky play-performance scales; SEN, special education needs.

## Discussion

The findings from this retrospective cohort study showed that one in three children with ABI had at least one seizure, and about one in four developed epilepsy. Compared to previous single-etiology studies—which reported that 17%−59% of children with brain tumors, TBI, stroke, ECMO, or CNS infections experienced seizures—our cohort demonstrated a 33% prevalence of post-ABI seizures epilepsy (31–34). Most acute seizures had focal onset (72%), often correlating with focal cortical injuries or non-cortical ABI complicated by focal intracerebral hemorrhage or obstructive hydrocephalus. Similarly, focal epilepsy accounted for 80% of cases, with most (60%) showing epileptiform discharges corresponding to the injured brain region. This pattern suggests that focal epileptogenesis may arise from neuronal and synaptic loss, aberrant sprouting with heterotropic neurons, astrogliosis, and rewiring, leading to permanent structural changes in the brain post-ABI (35). These observations align with previous studies indicating that acute seizures are associated with the underlying etiologies and sites of brain injury (36, 37).

In our cohort, 24% of patients were diagnosed with post-ABI epilepsy, with a median time to epilepsy onset of 2 years. Notably, one child developed epilepsy as late as 7.3 years post-ABI, indicating that the risk of epilepsy remains high even years after the initial injury. Regarding seizure control, half of the patients were seizure-free for more than 1 year, while the other half continued to experience ongoing seizures. Refractory epilepsy was observed in one-fourth of the cohort and was often associated with extensive parenchymal loss or encephalomalacia in the frontal or temporal regions. Although encephalomalacia alone may not directly cause epilepsy, the scarring of nearby brain tissue could lead to abnormal neuronal discharges, as the surrounding neuronal cells are pulled around the encephalomalacia, contributing to seizure generation (38). Diffuse brain damage (35) and focal encephalomalacia have been linked to pharmaco-resistant epilepsy (39), highlighting the importance of the extent and location of brain injury in prognosis and management.

Concerning the risk of seizures and epilepsy related to various etiologies and sites of ABI, we demonstrated that cortical injuries and CNS infections were associated with higher risk. This is consistent with existing study on early onset neonatal meningitis showing a 10-fold increased risk of epilepsy (40). Khan et al. also showed that the risk of unprovoked seizures remained high and tended to be recurrent 5 years after bacterial meningitis (41). In addition, we found that children who had seizures after a stroke had an elevated risk of subsequent seizure activity or epilepsy post-ABI. Existing studies demonstrated that the epilepsy risk post-stroke remained high compared to the general population even decades after ABI (6, 42). In children with brain tumors, we demonstrated that infratentorial tumors were correlated with reduced seizure recurrence, whereas existing study showed that cortical tumors were associated with higher seizure risk (31).

Two mechanisms have been proposed to explain why children with cortical injuries are more susceptible to developing epilepsy: (i) disinhibition of neural activity and (ii) the formation of new, functionally excitatory connections within the cortex. It has been proposed that structural cortical damage would lead to reduced production of brain-derived neurotrophic factor by pyramidal cells, resulting in atrophic changes in GABAergic interneurons and selective loss of inhibitory synapses, this would cause disinhibition of neuronal activities. Also, with increased excitatory cortical interneurons and pyramidal cells synaptic coupling, these lay groundwork for axonal sprouting within the injured area for new functional excessive recurrent hyperexcitable circuits (43). This hyperexcitability state has also been observed in children with CNS infections, which are thought to trigger an inflammatory response that disrupts the blood-brain barrier, contributing to increased neural excitability (44). Furthermore, in children with CNS infection, they are prone to intracranial bleeding, infarction and brain oedema, which could cause direct neuronal damage and subsequent glial scarring. Together, these could all lead to epileptogenesis (45).

In terms of seizure risk related to prophylactic anti-epileptic medications and other disease-specific therapies, we found no statistical difference in those given prophylactic AED compared to those without AED. A recent meta-analysis showed that AED prophylaxis seems to be effective against early posttraumatic seizures for the pediatric population but no observed benefit for late posttraumatic seizures (46), additional studies should be conducted to ascertain the benefits of prophylactic AED for children with non-traumatic ABI. In addition, we found that brain tumor patients receiving focal cranial irradiation were less likely to develop seizures. Our findings supported the use of targeted radiation techniques which might minimize brain tissue damage and reduce the risk of epileptogenesis. This underscores the importance of precise treatment planning to preserve healthy brain tissue, potentially lowering long-term neurological complications such as epilepsy.

Nearly all children (96%) in our study showed significant improvement in functional performance after intensive program-based rehabilitation training, with good improvement in functional outcomes at the latest follow-up compared to at ABI diagnosis. Our findings were consistent with previous study which showed that 70% of children had improved functional mobility after hospital-based rehabilitation (47). Chevignard et al. (48) also demonstrated the effectiveness of a structured ABI program for children and emphasized the importance of long-term organized and co-ordinated care for children with ABI. Future studies should also look at whether the intensity and duration of rehabilitation has an impact on functional outcomes.

More than half of our patients had borderline DQ/IQ or GDD/ID after ABI, and more patients in our seizure group required SEN support compared to the non-seizure group. Our findings were consistent with previous studies showing that children with a history of ABI had reduced academic achievement and greater educational needs following school re-entry (49, 50). Given the elevated risk of cognitive and learning difficulties in children with ABI, particularly those with seizures, incorporating cognitive rehabilitation into their overall rehabilitation program is essential. Early involvement of an educational psychologist to develop a personalized educational plan can facilitate timely academic support and promote successful school reintegration. This proactive approach can help optimize developmental and educational outcomes for these children.

## Limitations of our study

We acknowledge that our study has limitations with respect to its retrospective nature. Additionally, patients with relatively mild ABI such as concussion may not be referred to our local ABI programme, hence, may not be represented in our cohort. Although all our patients had significant ABI and the different follow-up times were adjusted statistically, we had a heterogeneous population with a relatively small subgroup sample size. We examined patients in various age ranges at ABI diagnosis with various etiologies, including different corresponding interventions and different prophylactic AED usage and durations. In addition, not all of our patients were monitored by EEG during their stay in pediatric intensive care units, hence, potential non-convulsive or electrographic seizures might not be documented.

## Conclusion

Our study described the seizure characteristics, onset, and outcomes in children with ABI. One-third of patients experienced post-ABI seizures. We found that CNS infection and cortical brain injuries were risk factors for post-ABI seizures. Although most children showed significant improvement in functional outcomes after the ABI, they are more likely to require extra support and special educational needs on returning to school.

## Data Availability

The raw data supporting the conclusions of this article will be made available by the authors, without undue reservation.

## Supplementary material

Supplementary Table S1 Individual characteristics of patients with post-ABI seizures.

Supplementary Table S2 Latest MRI Brain imaging of those with refractory epilepsy.

Supplementary Table S3 Types, dosages and durations of prophylactic AEDs given to the 25 patients with ABI.

Supplementary Table S4 Subscale IQ and DQ scores for children with ABI.
