# Supplementary material for: A retrospective cohort study on the seizure risks and outcomes of children with acquired brain injury
Source: Front Neurol. 2025 Sep 10;16:1629669. doi: 10.3389/fneur.2025.1629669 (PMC12459114; doi:10.3389/fneur.2025.1629669)
Supplement: Supplementary Table S1 — Individual characteristics of patients with post-ABI seizures. [file Table_1.docx]

Supplementary table 1. Individual characteristics for patients who have had seizures post ABI

| **#** | **Dx Age (Yr)** | **FU**  **(Yr)** | **Sex** | **ABI cause** | **ABI sites**  **If cortical: (Side), [Lobes]** | **Prophy-lactic AED (Months)** | **AS  (AED duration)** | **AS cause** | **AS**  **Type (onset)** | **Epilepsy**  **Type**  **(Onset)** | **Time to epilepsy (Yrs)** | | **SE Hx** | **Interictal EEG** | **Epilept-iform d/c same as ABI sites?** | **Current AED** | **Epilepsy control** |
| --- | --- | --- | --- | --- | --- | --- | --- | --- | --- | --- | --- | --- | --- | --- | --- | --- | --- |
|  |  |  |  |  |  |  |  |  |  |  | From ABI | From No AED |  |  |  |  |  |
| 1 | 0.92 | 6 | M | B (Rm) | M | N | Y  (LEV x 24m) | ICS (POD4) | G (TC) | N | / | / | N | / | / | / | / |
| 8 | 2.67 | 14 | F | B (Rm) | I | N | Y  (PB x 1.5m) | ICS (POD2) | Faw (Cl) | N | / | / | N | / | / | / | / |
| 9 | 4 | 7 | M | B (Rm) | I | N | Y (VPA x 1m &  LEV x 6m) | PS | Fim (Ton) | Fim (Ton)  [BTRE] | 0 | / | N | Not done | / | N | Free >12m |
| 12 | 11 | 4 | M | B (P) | C (R) [Fr,Pa] | N | Y (LEV till now) | PS | Faw (Cl) | Faw(Cl)  [BTRE] | 0 | / | N | ShW in Right Central | Y | LEV | Free >12m |
| 13 | 2.83 | 5 | M | B (Rm) | M | N | Y  (LEV x 0.3m) | ICS (POD0) | U | N | / | / | N | / | / | / | / |
| 16 | 0.42 | 7 | F | B (St) | C (Bil) [Fr,Pa] | N | Y  (PB x 16m & LEV x 14m) | PS | G (TC) | G (TC) [BTRE] | 0 | / | N | ShW in both hemispheres | Y | N | Free >12m |
| 17 | 0.83 | 5 | F | B (St) | C (R) [T] | N | Y (PB x 10m) | PS | Fim(Cl) | Fim (Cl)  [BTRE] | 0 | / | N | No epileptiform d/c | N/a | N | Free >12m |
| 21 | 6 | 2 | F | B (Rm) | C (L) [T] | N | Y  (OXC till now) | PS | Faw (Em) | Faw (Em) [BTRE] | 0 | / | N | ShW/ShSlW in left mid & posterior temporal | Y | OXC, CLB | DRE (Monthly seizures) |
| 22 | 1.75 | 5 | F | B (Rm) | C (L) [T] | N | Y (OXC x 48m) | PS | Fim (Em) | Fim (Em) [BTRE] | 0 | / | N | ShSlW in left side | Y | N | Free >12m |
| 25 | 0.33 | 3 | M | B (St) | C (R) [Fr] | Y  (LEV x 12m)  postop | N | / | / | G (TC) [BTRE] | 1.7 (BTRecur) | 0.7 | N | AW over bilateral occipital during sleep | N | LEV | Free >12m |
| 30 | 0.05 | 3 | M | H | D | N | Y (LEV x 6m) | H | F (Cl)^ | N | / | / | N | / | / | / | / |
| 42 | 7 | 7 | M | B (P) | I | N | N | / | / | Faw (Cl) [BTRE] | 7.3 (NewMet) | / | N | No epileptiform d/c | N/a | LEV | Last seizure 9m ago |
| 44 | 7 | 14 | F | S | D | Y  (VPA x 2days)  postop | Y (VPA x 1m) | ICS  (POD2) | Fim (Cl) | G (TC) | 4 | 3.9 | N | No epileptiform d/c | N/a | LCM, LEV | DRE (monthly seizures) |
| 45 | 0.21 | 7 | M | Tr | D | Y  (PHT x 1day) | Y  (PHT x 1m) | Tr | F (Cl)^ | N | / | / | N | / | / | / | / |
| 49 | 0.04 | 9 | F | In | M | N | Y  (PB x 5days) | In | F (Cl)^ | Faw  (Myo & Auto) | 3 | 3 | Y (E) | ESES | Y | STM | DRE (seizure free 11m after epilepsy Surgery) |
| 50 | 4 | 8 | M | B (Rm) | I | N | N | / | / | Fim (Cog) [BTRE] | 2 | / | N | no epileptiform d/c | N/a | N | Free >12m |
| 51 | 0 | 7 | M | In | C (Bil) [Fr,T] | N | Y (LEV x26m) | In+S | F (Cl)* | G (TC) | 2.6 | 0.4 | Y  (AS & E) | ShW/ShSlW in left side & right mid-posterior | Y | LEV | Free >12m |
| 62 | 0 | 6 | M | H | C (R) [O] | N | N | / | / | Fim (Cl) | 2.3 | / | N | ShW in Right occipital | Y | CBZ | Last seizure 1m ago |
| 66 | 0.17 | 5 | F | H | C (R) [Pa,O] | N | N | / | / | Fim (Auto) | 3 | / | N | ShSW & SW in left central-temporal region | N | N^^ | Last seizure 5m ago |
| 68 | 2.92 | 5 | M | H | D | N | N | / | / | Fim  (Ton, myo) | 2 | / | N | Bilateral multifocal SW & PSW, at times led to generalised d/c | Y | VPA^%^ | Daily seizure |
| 71 | 0.75 | 3 | F | Tr | C (L) [Pa] | N | Y (LEV x 1m) | HyperN | G (TC) | N | / | / | Y (AS) | / | / | / | / |
| 72 | 6 | 4 | M | S | C (Bil) [Fr] | Y (VPA x 6m)  postop | N | / | / | Faw (Ton) | 1.2 | 0.7 | N | SW over bifrontal | Y | VPA | Free >12m |
| 74 | 0.3 | 6 | F | In | D | N | Y (PB x 3m) | In | G (TC) | Faw (Cl) | 2.5 | 2.2 | Y (AS &E) | SW over R temporal | N | LEV | Last seizure 9m ago |
| 76 | 5 | 5 | F | S | D | Y (VPA x 1d postop) | Y (LEV x 13m) | S | Faw (Cl) | Faw (myo,aton) | 1.1 | AED has never been stopped | N | ShW & SW over both hemispheres | Y | VPA, CLB | DRE (Daily seizure) |
| 80 | 14 | 4 | M | In | C (R) [Fr] | Y (LEV x 2days) | Y (LEV x 9m) | ICS (POD2) | Faw (Cl) | N | / | / | N | / | / | / | / |
| 81 | 5 | 6 | M | B (Rm) | I | Y (VPA x 1m) postop | N | / | / | Fim (Ton,Aton,  Spasm, Beh) | 2.3 | 2.2 | N | SlSW most prominent over left mid-temporal, right posterior temporal when awake | Y^^^ | LEV, CLB, VGB, PB | DRE (Daily seizure) |
| 82 | 0 | 10 | F | B (Rm) | C (L) [Pa,O] | Y (VPA x 1wk) postop | N | / | / | Fim (Cl) | 4.2 | 4.2 | Y (E) | ShW/ShSlW in left hemispheres when drowsy & asleep | Y | LEV, VPA | Free > 12m |

Abbreviations: Dx, Diagnosis, FU, Follow-up time; F, Female; M, Male; AED, Anti epileptic drugs; AS, acute seizures within 7 days of ABI diagnosis; E, epilepsy; SE, status epilepticus seizure lasted >30mins; DRE, drug resistant epilepsy; Hx, history; B(Rm), brain tumour in remission; B(st), brain tumour with static residual tumour; B(P), brain tumour in palliative care; H, hypoxic brain injury; In, CNS infection; S, stroke; Tr, Traumatic brain injury; HyperN, hypernatremia; C, Cortical; R, right; L, Left; Bil, bilateral; Fr, Frontal; Pa, Parietal; T, Temporal; O, Occipital; Y, yes; N, No; PS, Presenting symptom; ICS; intracranial surgery; BTRecu, Brain tumour recurrence; NEwmet, New leptomeningeal spread; Faw, Focal seizures with preserved awareness; Fim, Focal seizures with impaired awareness, U, unknown onset (electrical seizures captured when patient was sedated & put on CFM) – no official EEG reporting; G (TC), Generalised tonic clonic seizures,

Cl = clonic; Aton = atonic; Ton= Tonic; TC= Tonic-clonic; Myo= myoclonic ; Cog = cognitive seizures; ShW = sharp waves ; ShSlW – sharp & slow waves; SW= spike waves ; PSW = polyspike waves

LEV – Keppra; OXC – Trileptal; CLB= clobazam ; PB = phenobarbitone

^Paitent 30 , 45, 49, 51– neonate/infant seizure (so no awareness category)

^^Patient 66 – not started on AED as very infrequent seizures and still pending further sleep EEG review (EEG only done when awake this time)

%Patient 68 – parents prefer not to step up AED ;

^^^Patient 81 - BT Dx 2017; Fragile X dx 2 years after brain tumour treatment – 2019, seizure dx 2020
