# Supplementary material for: A retrospective cohort study on the seizure risks and outcomes of children with acquired brain injury
Source: Front Neurol. 2025 Sep 10;16:1629669. doi: 10.3389/fneur.2025.1629669 (PMC12459114; doi:10.3389/fneur.2025.1629669)
Supplement: Supplementary Table S2 — Latest MRI Brain imaging of those with refractory epilepsy. [file Table_2.docx]

**Supplementary Table 2. Descriptive details of latest MRI Brain imaging of patients with refractory epilepsy.**

| Patient # | Latest MRI brain images of the patients with refractory epilepsy |
| --- | --- |
| 8 | Status post-resection of left temporal glioneuronal tumour. There is mild increased in T2/FLAIR signal along the resection margin, likely due to gliosis.  The mild ex-vacuo dilatation of temporal horn of left lateral ventricle is suggestive of parenchymal volume loss. |
| 13 | There is evidence of left parietal VP shunt with tip within the body of the right lateral ventricle. The ventricles are not dilated. Extensive area of encephalomalacia at the frontal lobes is similar, predominantly involving bilateral superior and middle frontal gyri. T2 and FLAIR hyperintensity. Similar cystic change is also seen at bilateral frontal corona radiata more on the left side, and left centrum semiovale. There is mild thinning and irregularity of the anterior portion of the corpus callosum. Findings likely relate to prior insults. Hypointense lesion at the pre-pontine cistern with susceptibility is in keeping with a known calcific lesion, appears similar in size, with no definite mass effect. Another focus of susceptibility seen just anterior to the right cerebral peduncle also corresponds to known calcific focus. Myelination pattern is otherwise within normal limits. Grey-white matter differentiation appears within normal limits. A focus of susceptibility is seen abutting the septum pellucidum at the body of the right lateral ventricle, could represent haemosiderin deposition from previous haemorrhage. |
| 15 | Cystic encephalomalacia changes at right frontal, right parietal and right occipital regions are similar, showing communication with right lateral ventricle, which shows ex-vacuo dilatation. Atrophic changes noted over right lentiform nucleus, caudata, and thalamus. Wallerian degeneration of right cerebral peduncle noted. Abnormal high T2W signal noted over left thalamus. Imaging findings are in keeping with prior insult.  Previously noted serpentine high T1W signal at extra-axial right high parietal and parasagittal right occipital regions are not well seen. |
| 24 | Established patchy areas of cystic encephalomalacia involving the cerebral cortices, most significant over bilateral frontal, bilateral parietal, and bilateral temporal lobes, overall, left side worse than right. Abnormal gliotic signal changes are also similarly seen in bilateral lateral cerebellar hemispheres and cerebellar vermis and associated with mild cerebellar atrophy. |
| 26 | There are post-operative changes at the left temporal lobe and cerebellar hemisphere, and linear enhancing focus along the superior margin of left temporal operative site. These are similar to previous scan. There are multiple bilateral periventricular cystic changes that are similar. Dilated ventricular system, similar in morphology and extent as compared to previous scan. Bilateral subdural collections. Diffuse pachymeningeal thickening are also similar to previous scan. New nodular lesion at posterior aspect of thecal sac at level of L3. Suspected patchy enhancement mingled with cauda equina nerve roots at levels L2 and L3. These were not seen previously. New spinal drop metastases have to be considered in this clinical setting. Smallish spinal canal from L2 downwards (likely developmental) with cauda equina crowding. |
