# Supplementary material for: A retrospective cohort study on the seizure risks and outcomes of children with acquired brain injury
Source: Front Neurol. 2025 Sep 10;16:1629669. doi: 10.3389/fneur.2025.1629669 (PMC12459114; doi:10.3389/fneur.2025.1629669)
Supplement: Supplementary Table S3 — Types, dosages and durations of prophylactic AEDs given to the 25 patients with ABI. [file Table_3.docx]

**Supplementary table 3. Types, Dosages and Duration of Prophylactic AEDs given to the 25 patients with ABI.**

| **Patient number** | **Age at diagnosis (years)** | **Gender (F/M)** | **Types of ABI** | **Prophylactic AED** | **Prophylactic AED given** | **AED Dosage** | **Duration of prophylactic AED** | **Acute Seizure within 7 days of diagnosis of ABI** | **Epilepsy** | **Time (years) from diagnosis to seizure onset** | **Current AEDs** | **Refractory epilepsy? (needing >2 AEDs)** |
| --- | --- | --- | --- | --- | --- | --- | --- | --- | --- | --- | --- | --- |
| 1 | 4.0 | F | B | Y | Phenytoin | 20mg/kg/dose | 1 dose | N | N | / | / | / |
| 2 | 2.8 | F | B | Y | Levetiracetam | 20mg/kg/day | 1 week | N | N | / | / | / |
| 3 | 1.4 | M | B | Y | Levetiracetam | 20mg/kg/day | 2 weeks | N | N | / | / | / |
| 4 | 1.7 | F | B | Y after first surgery | Phenytoin | 10mg/kg/day | 3 weeks | N | N | / | / | / |
|  |  |  |  | Y after second surgery | Valproate | 10mg/kg/day | 2 weeks | - | - | - | - | - |
| 5 | 5.0 | M | B | Y | Levetiracetam | 15mg/kg/day | 3 weeks | N | N | / | / | / |
| 6 | 7.0 | M | B | Y | Valproate | 20mg/kg/day | 4 weeks | N | N | / | / | / |
| 7 | 6.0 | F | B | Y | Levetiracetam | 24mg/kg/day | 2 months | N | N | / | / | / |
| 8 | 4.0 | M | B | Y | Levetiracetam | 18mg/kg/day | 1 month | N | N | / | / | / |
| 9 | 0.2 | M | B | Y after first surgery | Levetiracetam | 15mg/kg/day | 3 months | N | N | / | / | / |
|  |  |  |  | Y after second surgery | Levetiracetam | 24mg/kg/day | 2 months | - | - | - | - | - |
| 10 | 0.0 | F | B | Y after first surgery | Levetiracetam | 20mg/kg/day | 3 months | N | N | / | / | / |
|  |  |  |  | Y after second surgery | Levetiracetam | 10mg/kg/day | 3 months | - |  |  |  |  |
| 11 | 5.0 | M | B | Y | Valproate | 30mg/kg/day | 2 months | N | N | / | / | / |
| 12 | 4.0 | M | B | Y | Valproate | 10mg/kg/day | 2 weeks | N | N | / | / | / |
| 13 | 9.0 | M | Tr | Y | Phenytoin | 5mg/kg/day | 1 week | N | N | / | / | / |
|  |  |  |  | Immediately followed Phenytoin | Valproate | 25mg/kg/day | 3 weeks | - | - | - | - | - |
| 14 | 5.0 | F | B | Y | Phenytoin | 5mg/kg/day | 4 weeks | N | N | / | / | / |
| 15 | 10.0 | F | B | Y | Valproate | 30mg/kg/day | 1 month | N | N | / | / | / |
| 16 | 0.2 | M | B | Y | Phenobarbitone | 5mg/kg/day | 1 week | N | N | / | / | / |
| 17 | 6.0 | M | S | Y | Valproate | 25mg/kg/day | 3 weeks | N | N | / | / | N |
| 18 | 0.3 | M | B | Y | Levetiracetam | 40mg/kg/day | 11 months | N | Y | 1.7 | Levetiracetam | N |
| 19 | 7.0 | F | S | Y | Valproate | 18mg/kg/day | 1 month | Y | Y | 4 | Lacosamide & Levetiracetam | Y |
| 20 | 0.2 | M | Tr | Y after first surgery | Phenytoin | 5mg/kg/day | 1 month | Y | N | / | N | N |
|  |  |  |  | Y after second surgery | Valproate | 10mg/kg/day | 1 week | - | - | - | - | - |
| 21 | 6.0 | M | S | Y | Valproate | 15mg/kg/day | 6 months | N | Y | 1.2 | Valproate | N |
| 22 | 5.0 | F | S | Y | Valproate | 20mg/kg/day | 3 months | Y | Y | 1.1 | Valproate & Clobazam | Y |
| 23 | 14.0 | M | In | Y | Levetiracetam | 20mg/kg/day | 6 months | Y | N | / | N | N |
| 24 | 5.0 | M | B | Y | Valproate | 30mg/kg/day | 2 weeks | N | Y | 2.3 | Levetiracetam, Clobazam, Vigabatrin, Phenobarbitone | Y |
| 25 | 0.0 | F | B | Y | Valproate | 30mg/kg/day | 1 week | N | Y | 4.2 | Levetiracetam & Valproate | N |

Abbreviation: ABI, acquired brain injuries; AED, anti-epileptic drugs; B, Brain tumours; F, Female; In, CNS infection; M, Male; ; N, No; S, Stroke, Tr, Trauma; Y, Yes
