# Supplementary material for: A retrospective cohort study on the seizure risks and outcomes of children with acquired brain injury
Source: Front Neurol. 2025 Sep 10;16:1629669. doi: 10.3389/fneur.2025.1629669 (PMC12459114; doi:10.3389/fneur.2025.1629669)
Supplement: Supplementary Table S4 — Subscale IQ and DQ scores for children with ABI. [file Table_4.docx]

To better understand the impact of seizures, Wilcoxon rank-sum tests were used to compare groups. Among ABI cases aged ≥ 6 years, there were no significant differences in any IQ subdomains between those with and without seizures (all p > 0.05). Likewise, in children aged ≤ 6 years, seizure status was not associated with significant differences in either Motor outcomes, speech outcomes abilities or executive function—measured by the Griffiths Scales of Child Development (all p > 0.05), see Table S3.

Table S3 Outcomes in children with ABI.

|  | Total | Non-seizure | Seizure | p value^4^ |
| --- | --- | --- | --- | --- |
| Full scale IQ score (>6yo)^1^ | 82.5 (27.5) | 87.0 (25.0) | 77.0 (28.5) | 0.225 |
| Perceptual Reasoning Index | 89.0 (28.0) | 92.0 (24.8) | 77.0 (34.0) | 0.196 |
| Processing Speed Index | 83.0 (21.0) | 82.0 (22.2) | 83.0 (15.0) | 0.761 |
| Working Memory Index | 91.0 (20.0) | 91.0 (20.8) | 85.0 (18.0) | 0.855 |
| Verbal Comprehension Index | 92.0 (19.0) | 94.0 (19.5) | 84.0 (20.5) | 0.306 |
| DQ score (<6yo)^2^ | 73.0 (40.8) | 71.0 (35.0) | 75.0 (63.0) | 0.338 |
| Motor outcome | 60.0 (38.0) | 60.0 (25.0) | 43.0 (69.5) | 0.650 |
| Hearing and Speech outcomes | 59.0 (28.0) | 59.0 (20.0) | 59.0 (34.0) | 0.633 |
| Executive Function^3^ | 74.2 (26.8) | 71.0 (19.0) | 77.5 (49.0) | 0.614 |

1. IQ, intelligence quotient
2. DQ, development quotient
3. Estimated using Griffiths Scales of Child Development E(Performance) and F(Practical Reasoning)
4. P values are calculated based on Wilcoxon rank-sum tests
